# Indian Long-term Non-Progressors Show Broad ADCC Responses with Preferential Recognition of V3 Region of Envelope and a Region from Tat Protein

**DOI:** 10.3389/fimmu.2017.00005

**Published:** 2017-01-19

**Authors:** Archana Kulkarni, Swarali Kurle, Ashwini Shete, Manisha Ghate, Sheela Godbole, Vijaya Madhavi, Stephen J. Kent, Ramesh Paranjape, Madhuri Thakar

**Affiliations:** ^1^Department of Immunology and Serology, National AIDS Research Institute, Pune, India; ^2^Department of Clinical Sciences, National AIDS Research Institute, Pune, India; ^3^Department of Epidemiology and Biostatistics, National AIDS Research Institute, Pune, India; ^4^Department of Microbiology and Immunology, Peter Doherty Institute for Infection and Immunity, University of Melbourne, Melbourne, VIC, Australia

**Keywords:** LTNP, NK cells, ADCC, envelope, Tat

## Abstract

HIV-specific antibody-dependent cell cytotoxicity (ADCC) is likely to be important in governing protection from human immunodeficiency virus (HIV) and slowing disease progression. Little is known about the ADCC responses to HIV-1 subtype C. We characterized ADCC responses in HIV-1 subtype C-infected Indian subjects with slow disease progression and identified the dominant antigenic regions recognized by these antibodies. ADCC responses were measured in plasma from 34 long-term non-progressors (LTNPs), who were asymptomatic and maintained CD4 count above 500 cells/mm^3^ for the last 7 years in the absence of antiretroviral therapy (ART), and 58 ART naïve progressors with CD4 count <500 cells/mm^3^ against overlapping HIV-1 peptides using a flow cytometry-based antibody-dependent natural killer (NK) cell activation assay. The assay measured CD107a expression on NK cells as a marker of antibody-dependent NK cell activation and IFN-γ secretion by NK cells upon activation. The ADCC epitopes were mapped using the matrix of overlapping peptides. Indian LTNPs showed higher and broader ADCC responses compared to the progressors. The Env-C and Tat-specific ADCC responses were associated with lower plasma viral load, whereas the Env-C responses were also associated with higher CD4 counts. Five of 10 LTNP responders targeted epitopes in the V3 region (amino acids 288–330) of Env-C. Additionally, three Tat regions were targeted by ADCC antibodies from LTNPs. ADCC responses were associated with slow HIV progression in Indian subtype C-infected cohort. The frequently recognized peptides from the V3 loop of Env and the novel epitopes from Tat by the LTNPs warrants further study to understand the role of ADCC responses to these regions in control and prevention of HIV-1 infection.

## Introduction

A vaccine is urgently needed for ultimate success in controlling human immunodeficiency virus (HIV) epidemic. However, one of the major obstacles in this endeavor is that the correlates of immunity to HIV are poorly understood. Fc-mediated functions of Env-specific antibodies, such as antibody-dependent cell cytotoxicity (ADCC), correlated with protection against HIV infection in an RV 144 phase III HIV vaccine trial (1). Further, ADCC responses have been associated with protection against infection in newborns (2). However, data are scarce on the ADCC responses in the individuals infected with different HIV subtypes especially infected with Indian strains of HIV-1.

Human immunodeficiency virus-infected individuals who are able to maintain CD4 counts above 500 cells/mm^3^ for extended time period, usually more than 7 years of infection in the absence of antiretroviral treatment (ART) are commonly termed as long-term non-progressors (LTNPs) (3). Although a subset of LTNPs generates broadly neutralizing antibodies or efficient T cell immunity to HIV, such immune responses do not fully explain their slower disease progression (4–6). Fc-mediated antibody responses can mediate clearance of infected cells and may contribute to slower disease progression (7). However, these responses have not been characterized well in subtype C-infected Indian subjects. Further, the information on HIV epitopes recognized by ADCC antibodies across different HIV clades is limited.

We have previously shown that the natural killer (NK) cells from Indian LTNPs are functionally competent in lysing HIV-infected cells (8). However, the information about ADCC-mediating antibodies in these patients was not known. Recent advances in assays to measure the ability of ADCC antibodies to activate NK cells in response to pools of overlapping HIV-1 peptides (9) allowed us to characterize linear peptide ADCC epitopes recognized by HIV subtype C-infected Indian subjects. This study was undertaken to estimate and characterize the ADCC responses for their magnitude and breadth in Indian LTNPs and to identify the epitopes recognized by them.

## Materials and Methods

### Study Participants

Study participants were identified among the attendees of the outpatient clinics of the National AIDS Research Institute who visited the clinic regularly for care and support. LTNPs were identified as individuals with asymptomatic HIV infection for more than 7 years who had stable CD4 counts above 500 cells/μl and who had not taken ART (3). Progressors were identified as ART-naive HIV-infected patients with CD4 counts <500 cells/μl. Thirty-four LTNPs and 58 progressors were enrolled in the study along with 14 HIV-uninfected healthy individuals. Demographic, immunological, and virological characteristics of 92 HIV+ subjects are shown in Table 1.

**Table 1:** **Demographic, immunological, and virological characteristics of human immunodeficiency virus-infected study participants**.

	Long-term non-progressor	Progressors
Number of subjects	34	58
Median age (years)	38.5	35
IQR	(35–45)	(31–39)
Male/female	11/23	34/24
Years of seropositivity (mean and range)	10.8 years (7–19 years)	–
CD4 count at the testing visit (cells/mm^3^) [median and IQR]	699 (632–942)	409 (315–456)
	Mann–Whitney *U*-test *p* < 0.0001	
Plasma viral load at testing visit (RNA copies/ml) [median and IQR]	2,174 (422–9,799)	17,148 (5,543–87,100)
	Mann–Whitney *U*-test < 0.0003	
Number of viremic controllers (viral load <2,000 RNA copies/ml)	12	0
Number of non-viremic individuals (viral load >2,000 RNA copies/ml)	22	58

The study was approved by the institutional ethical committee. Participants were enrolled after obtaining written informed consent.

### ADCC Intracellular Cytokine Staining Assay (NK Cell Activation Assay)

**Figure 1:**
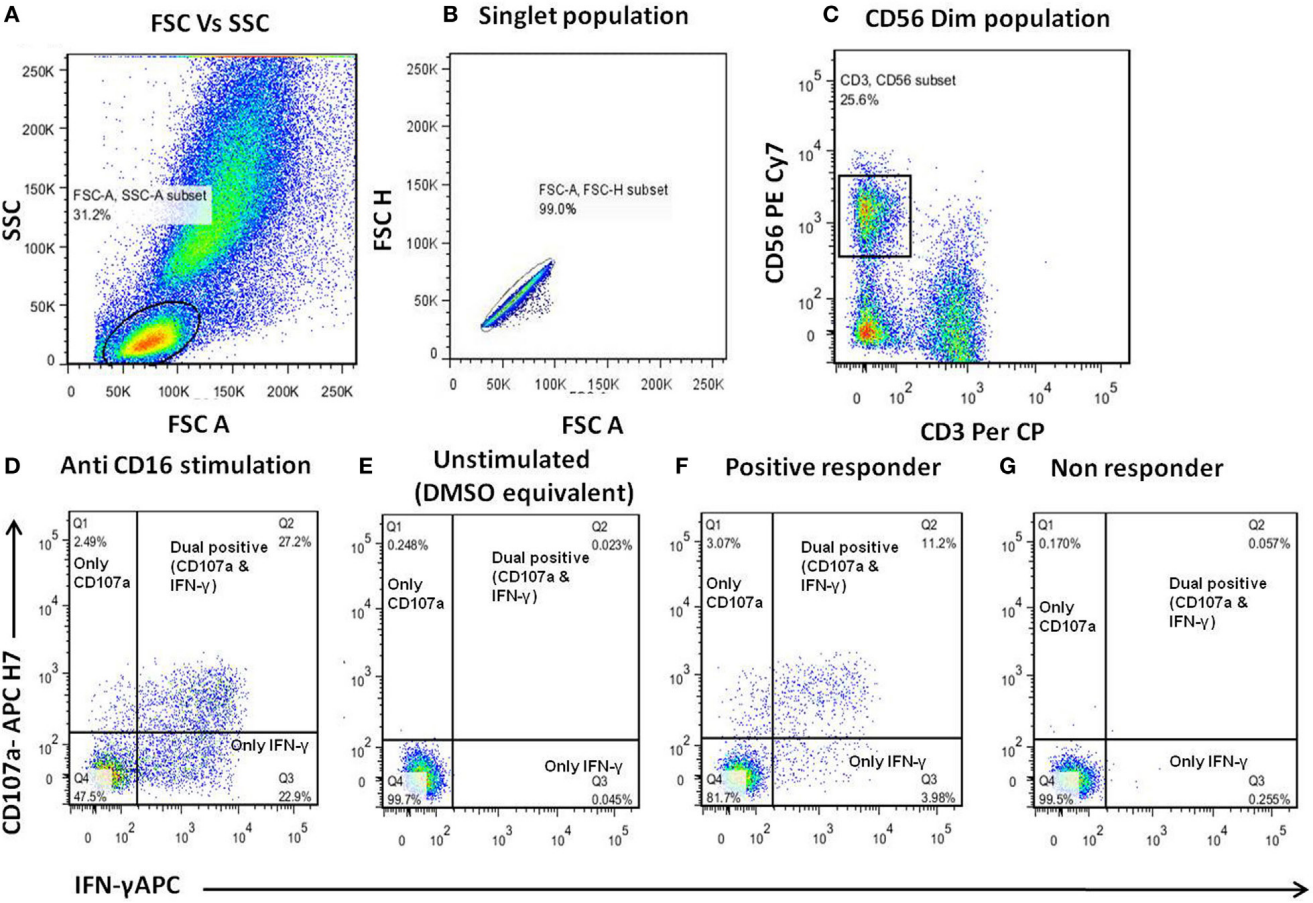
**Gating strategy for antibody-dependent natural killer (NK) cell activation assay**. **(A–C)** Donor blood (as a source of NK cells) was incubated with the serum/plasma test samples and human immunodeficiency virus (HIV) peptide pools for 5 h and then analyzed by flow cytometry. The lymphocytes were gated using FSC/SSC scatter. The NK cells were identified as CD3−CD56+ cells and assessed for surface CD107a expression and intracellular IFN-γ expression. **(D)** Representative dot plots for positive control (purified anti-CD16). **(E)** Unstimulated (DMSO equivalent solution) control. **(F,G)** Representative displays of ADCC responder (patient-1) and non-responder (patient-2) to HIV-1 Env-C peptide pool.

The ADCC response in the serum/plasma samples of the study participants was determined using NK cell activation assay as described previously (10). The assay uses CD107a and IFN-γ expression by NK cells as a final readout. We preferred this assay, although it does not measure the killing of infected target cells, as this assay was helpful in allowing us to map the linear epitopes recognized by ADCC-mediating antibodies and also captured cytokine secretion (IFN-γ), an another important aspect of antibody-mediated NK cell activation. Also, it has been recently shown that the ADCC activity observed in NK cell activation assay correlates significantly with functional killing assays such as the rapid fluorometric ADCC assay (11) and other assays (12–17). The anti-HIV ADCC responses were assessed against consensus envelope [both HIV-1 B and C], Gag (HIV-1C consensus), Pol (HIV-1 B consensus), and accessory proteins: Nef, Vpu, Rev, and Tat (HIV-1 B consensus) using pools of 15mer peptides overlapping by 11aa (National Institutes of Health AIDS reagent program). The detailed description of the peptides and the procedure for preparation of peptide pools is given in Supplementary Material. Briefly, 150 µl whole blood from healthy donor (as a source of NK cells) and 50 µl of heat-inactivated heparin-anticoagulated plasma or serum [final concentration as1:4] from the study participants was incubated at 37°C with HIV peptide pools (at a final concentration of 1 μg/ml/peptide) for 5 h in the presence of brefeldin A (10 μg/ml) (Sigma) and monensin (0.68 μl/ml) (BD Biosciences). After incubation, CD3−CD56dim NK cells were gated and analyzed for the expression of intracellular IFN-γ and CD107a using fluorescent tagged antibodies [CD3 Per Cp: (clone-SK7), CD56 PE-Cy7: (clone-HCD56), and CD107a APC H7: (clone-H4A3) (all from Biolegend), and IFN-γ APC: (clone-B27) (BD Biosciences)]. The gating strategy is shown in Figures 1A–C. Stimulation by purified anti-CD16 monoclonal antibody (clone-3g8, at 2.5 μg/ml concentration) was used as a positive control (Figure 1D). Plasma samples of all study groups incubated with HIV-negative donor blood in the absence of antigen was considered as a negative control for the respective sample. Mock control containing plasma, HIV-negative donor blood, and DMSO equivalent solution was used to negate the effect of DMSO that was used to dissolve peptides on NK cell activation (Figure 1E). The percentage of activated NK cells as a marker of ADCC activity was represented as the sum of the percentage of any activated NK cells, expressing only CD107a, both CD107a and IFN-γ and only IFN-γ (Figures 1F,G). The percent NK cell activation seen in mock stimulation (donor blood (NK cells) and test plasma incubated along with DMSO equivalent solution) for each sample was subtracted from this sum. The NK cell activation in mock control (plasma+ cells without stimulation) ranged from 0.124 to 4.41% with the mean of 1.48%. The response was considered to be positive if the following two criteria were fulfilled: (1) the total percentage of CD107a and IFN-γ expressing (activated) NK cells was more than three times the percentage of activated NK cells from the negative control of the respective sample and (2) the total percentage of activated NK cells was greater than the mean plus two SD of the percent activated NK cells from 14 HIV-negative donors tested for each of the HIV antigens. The interassay variation was found to be <10% as assessed by determining the % CV for positive control [Anti-CD16 stimulation] was 7.18% and negative control [donor blood (source of NK cells) without any stimulant] was 9.8%.

### Mapping of Epitopes Recognized by ADCC Antibodies

The samples of ADCC responders were further used to identify linear peptides recognized by ADCC antibodies using the peptide matrix pools in NK cell activation assay. The matrix pools were designed in such a way that each peptide is present in two different pools as described previously (18). The peptide that was common in two pools showing that ADCC response was identified as the peptide recognized by ADCC antibodies and the recognition of the particular peptide was further confirmed by repeating the NK cell activation assay using the single peptide. For mapping the epitopes, 30 pools for HIV-1 subtype C Env peptides and 10 pools for HIV-1 subtype B Tat peptides were used. Details of the pools are given in Figures S1A,B in Supplementary Material.

### Statistical Analysis

Statistical analyses were performed using GraphPad Prism, version 5.0 (GraphPad Software, San Diego, CA, USA). Data were analyzed by Mann–Whitney *U* test to compare the magnitude of ADCC responses between LTNP and progressor groups. Correlation analyses were performed using Spearman correlation between CD4 count, viral load, and magnitude of ADCC responses. Fisher’s exact test and chi square tests were used to analyze the significance of ADCC responses in different categories.

## Results

### LTNP and Progressor Cohorts

Thirty-four HIV-infected individuals who fulfill criteria for LTNP were enrolled in the study. As a comparator, we selected 58 progressor subjects with CD4 T cell counts of <500/μl. The median age of LTNPs (median 39 years [IQR 35–45]) and progressors (median 35 [IQR 31–39]) was not significantly different. Of the 34 LTNPs, 23 were female and 11 were male, versus 24 and 34 among the 58 progressors, respectively. The mean seropositivity in LTNP cohort was 10.8 years with a range from 7 to 19 years. The CD4 counts of LTNPs at the study visit (median 699 cells/μl [IQR 632–942]) were significantly higher than in the progressors (median 409 cells/μl [IQR 315–456]) (*p* < 0.001). As expected, the plasma viral load values at the study visits were significantly lower in LTNPs (median 2,174 HIV RNA copies/ml [IQR 422–9,799]) when compared to the values from progressors (median 17,148 [IQR 5,543–87,100]) (*p* = 0.0002) (Table 1).

### Indian LTNPs Have Significantly Higher Levels of ADCC Responses

**Figure 2:**
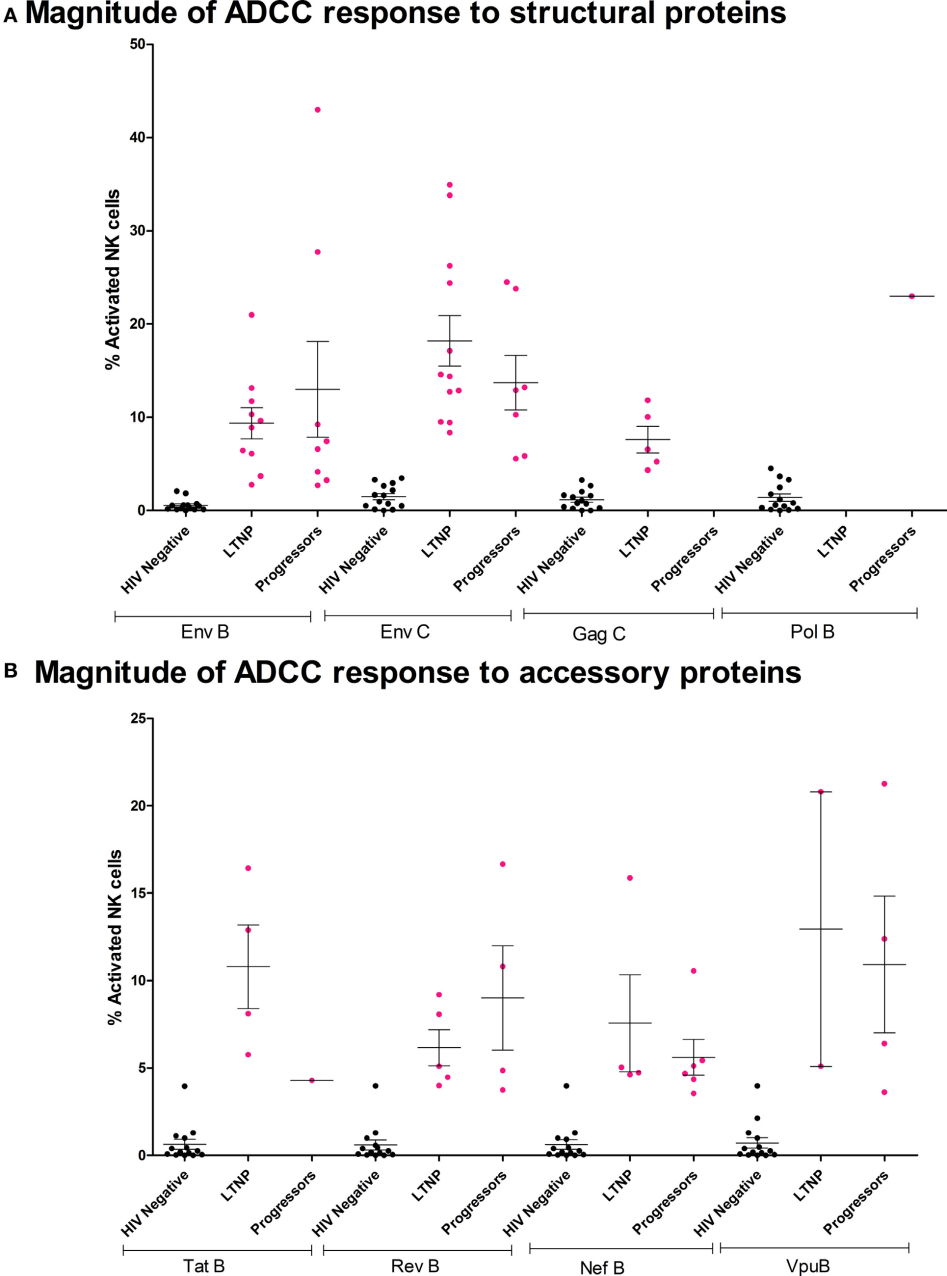

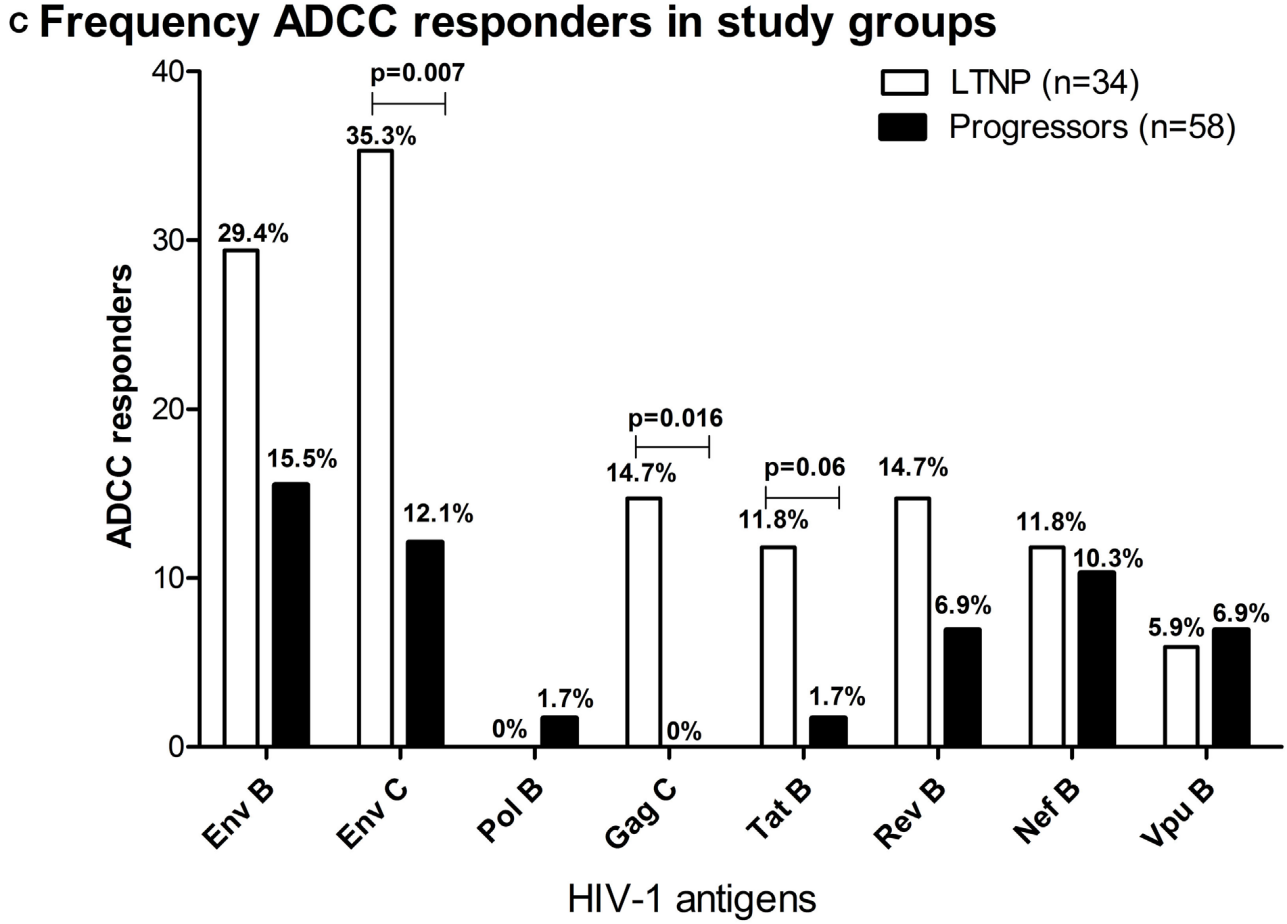
**Characteristics of ADCC responses in Indian long-term non-progressors (LTNPs) and progressors**. Vertical scatter plots (error bars—mean with SEM) denote magnitudes of ADCC-mediated natural killer (NK) cell activation in responders from LTNPs and progressors against HIV-1 antigen peptide pools. Magnitude of ADCC response against **(A)** structural proteins: HIV-1 B Env, HIV-1 C Env, HIV-1 C Gag, and HIV-1 B Pol and **(B)** accessory proteins: HIV-1 B Tat, Rev, Nef, and Vpu. The magenta colored dots represent ADCC responders (fulfilled both criteria mentioned in Materials and Methods section). The non-specific recognition of the peptides as indicated by the % NK cell activation by HIV-negative plasma after stimulation by same peptide pools is indicated in black dots. **(C)** Bar diagram represents the frequency of responses in both cohorts against HIV-1 B and C Env, HIV-1 C Gag, HIV-1 B Pol, Tat, Rev, Nef, and Vpu. The frequencies were higher in LTNPs in case of responses against Env subtype C and Gag, whereas a trend of higher tat-specific responses was observed in LTNPs although not significant (Fisher’s exact 2 × 2 test).

A total of 20 of 34 LTNPs (58.8%) and 20 of 58 progressors (34.4%) showed antibody-dependent NK cell activation against at least one of the HIV overlapping peptide pools. The trend of higher magnitude in LTNP was observed in the case of Env-C [LTNP mean 18.2%; range 8.4–35%, progressors mean 13.72%; range 5.6–24.5%], Tat B [LTNP mean 10.8%; range 5.8–16.4%, progressors mean 4.3%], Nef B [LTNP mean 7.6%; range 4.6–15.87%, progressors mean 5.6%; range 3.5–10.6%], and Vpu [LTNP mean 13%; range 5.1–20.8%, progressors mean 11%; range 3.6–21%]. Whereas a trend of higher magnitude in progressors was observed in the case of Env B (LTNP mean 9.36%; range 2.8–21%, progressors mean 13.01%; range 2.7–43%) and Rev B [LTNP mean 6.2%; range 4–9.2%, progressors mean 9%; range 3.7–16.6%]. In the case of Gag C, ADCC responses were seen only in the LTNP cohort and only one from progressor group showed the ADCC response against Pol B peptide pool (Figures 2A,B). The number of LTNPs showing Env-C and Gag-specific ADCC responses was also significantly higher than among progressors (*p* < 0.007 and *p* < 0.016, respectively). The number of responders against Tat antigen was also higher in LTNPs, but the difference did not reach significance (*p* = 0.06, Fisher’s 2 × 2 exact test), whereas the number of responders against HIV-1 subtype B Env peptides, Pol, Nef, Rev, and Vpu antigens was similar in both groups (Figure 2C). Although the magnitude of ADCC response is shown as % total NK cell activation (sum of % NK cells expressing CD107a and/or IFN-γ), overall the distribution of CD107a and IFN-γ expression of activated NK cells by the NK cell-activating antibodies within the plasma of the LTNPs and progressors was found to be skewed toward higher IFN-γ expression in both groups (Table S2 in Supplementary Material).

**Figure 3:**
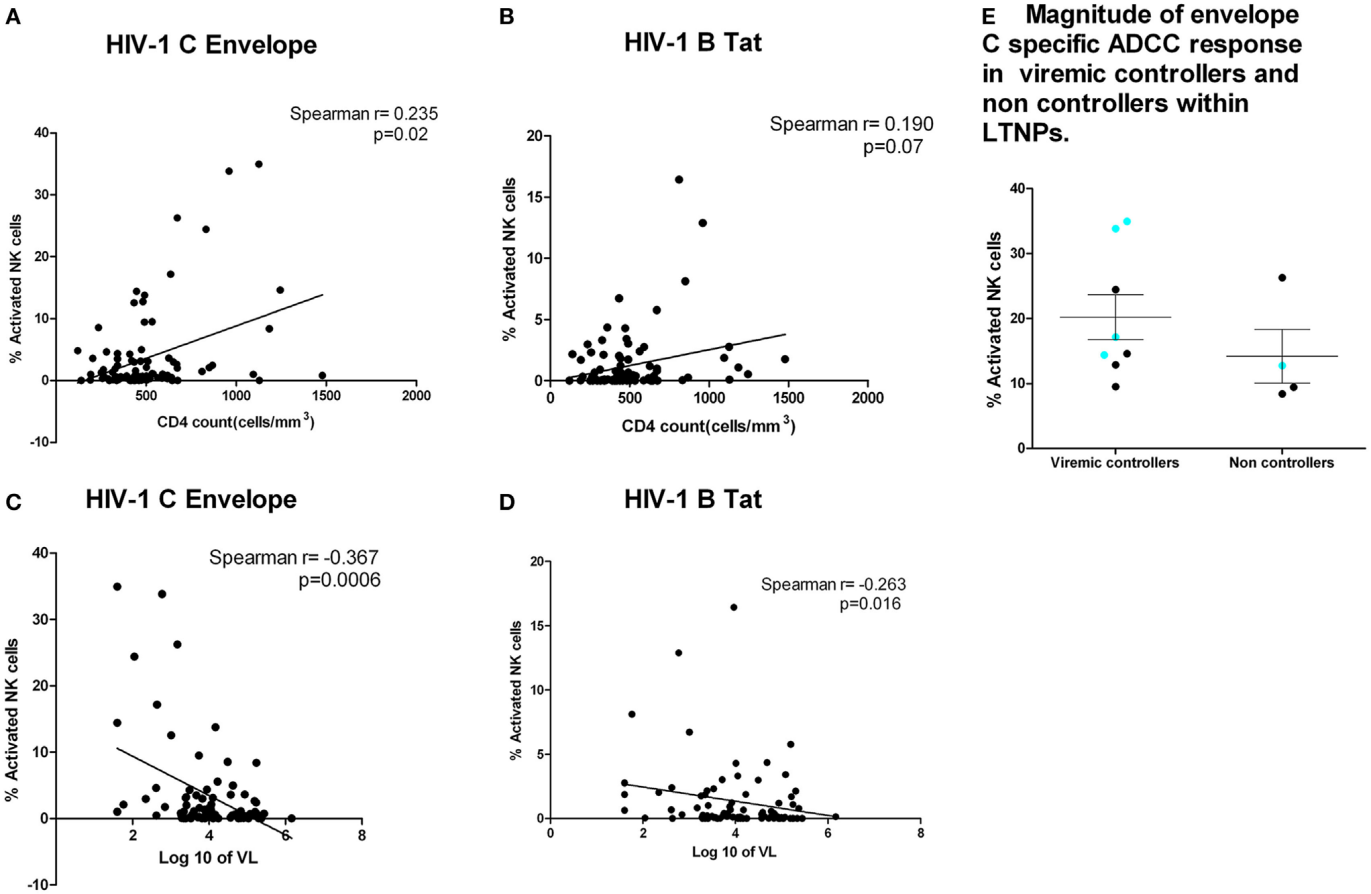
**ADCC responses were associated with higher CD4 count and lower plasma viral load**. CD4 counts at the testing visit (*N* = 92) (*X* axis) with envelope C peptide pool (*Y* axis) (*p* = 0.02) **(A)** and Tat-specific ADCC responses (*Y* axis) (*p* = 0.07) **(B)**. **(C,D)** Association of plasma viral load at the testing visit (*N* = 92) with ADCC response against Env-C peptide pool and Tat B peptide pool, respectively. **(E)** Magnitude of envelope C-specific ADCC response in viremic controllers and non-controllers within long-term non-progressors (LTNPs). The magnitude of ADCC responses [% natural killer cell activation] was comparable in between the groups, but the number of responders to envelope C was high in viremic controllers (8 out of 12) (plasma viral load <2,000 copies/ml) as against 4 out of 22 LTNPs with plasma viral load >2,000 copies/ml. Additionally, the preferential recognition of V3 region (aa 288–330) as indicated by blue dot was seen in four out of eight responders from viremic controller LTNPs as against only one out of four responders from non-controllers.

We then analyzed whether the magnitude of the Env subtype C, Tat, and Gag-specific ADCC responses in the HIV-infected individuals correlated with CD4 T cell count and viral load; standard markers of HIV disease progression. Anti-Env ADCC responses were modestly correlated with CD4 T cell counts (*r* = 0.235, *p* = 0.02, Figure 3A). There was a trend toward higher CD4 T cell counts in subjects with higher Tat-specific ADCC responses (Spearman = 0.190, *p* = 0.07, Figure 3B). The plasma viral load values were negatively correlated with ADCC responses against Env-C specific ADCC (*r* = −0.367, *p* = 0.0006, Figure 3C) and Tat (*r* = −0.263, *p* = 0.016, Figure 3D) antigens. Also, a significant association between Env-C ADCC responses and lower plasma viral load was observed when only LTNP group was separately analyzed (Spearman *r* = −0.377; *p* = 0.04). Besides this, the median plasma viral load values (calculated based on annual viral load values obtained during the follow-up period) among LTNPs were also negatively correlated with ADCC responses against Env-C peptide pool (*r* = −0.505, *p* = 0.004). There was no significant correlation between CD4 T cell counts or viral load values with the magnitude of Gag-specific ADCC responses (data not shown). Our definition of LTNPs was immunological (preserved CD4 T cell counts for at least 7 years) rather than virological. Hence, to further study the role of Env-specific ADCC in control of viral load, we divided the LTNP cohort into those with low-level viremia (median VL load <2,000 RNA copies/ml during follow-up) and with higher viral loads (median >2,000 RNA copies/ml). The magnitude of ADCC response against envelope C peptide pool was similar in the viremic controllers and non-controllers within LTNP (*p* = 0.15). Out of five responders to V3 loop epitope from LTNP, four were viremic controller subgroup as against only one was from non-controller (Figure 3E).

### The ADCC Responses in LTNPs Have Higher Breadth

**Figure 4:**
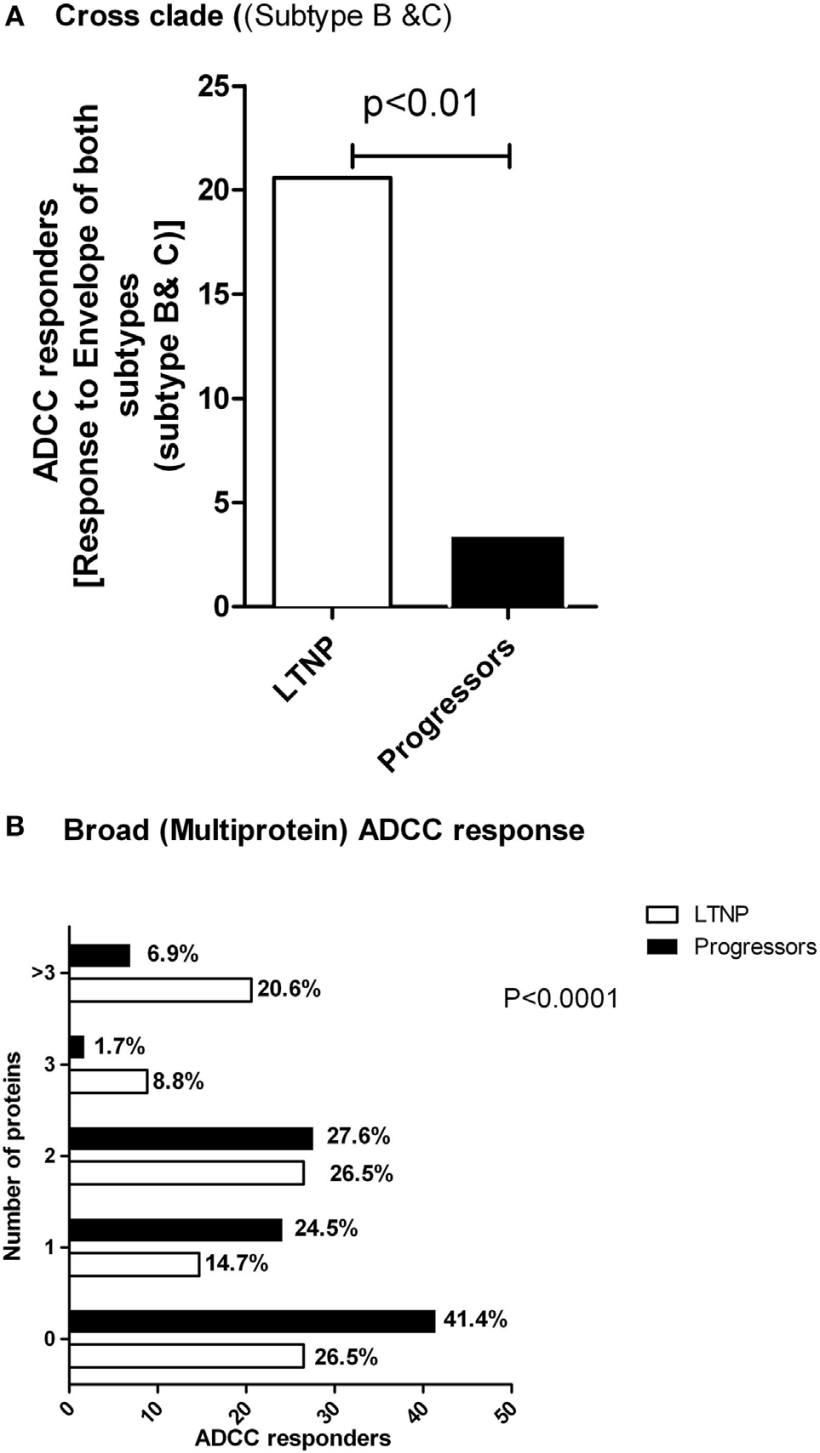
**LTNPs showed cross clade as well as broad ADCC responses**. **(A)** Percentage of the responders (*Y* axis) to both Env-C and -B peptides is significantly higher in LTNPs as compared to the progressors (*p* = 0.01 in a 2 × 2 Fischer’s exact test). **(B)** The breadth of the ADCC response. The number of human immunodeficiency virus antigens (*Y* axis) recognized by ADCC antibodies from LTNPs were significantly higher than the number of antigens recognized by progressors (chi square test, *p* < 0.0001).

The ability of ADCC responses to recognize diverse HIV strains is likely to improve the effectiveness of ADCC antibodies as a prevention strategy. Hence, to study the breadth of the ADCC antibody specificities, we analyzed whether LTNPs and progressors recognized both subtype C and B Env peptide pools. We found that of the 34 LTNPs, 20.6% (*N* = 7) had ADCC responses against both HIV-1 C and B Env peptides, whereas only 3.4% (2/58) of the progressors showed response against both B and C Env peptides (*p* < 0.01 by Fisher’s 2 × 2 exact test, Figure 4A).

Combinations of broad ADCC responses might also contribute to slow HIV progression given our findings on Env- and Tat-specific ADCC responses as mentioned above. To study this, we analyzed both the cohorts that had positive ADCC responses to 0, 1, 2, 3 or >3 of the HIV antigens represented by peptide pools. We found that the ADCC responses in the LTNPs showed significantly greater breadth compared to the progressors. A total of 21% of LTNPs showed responses against more than three antigens, compared to 7% in progressors (chi square test, *p* < 0.0001, Figure 4B).

### Indian LTNPs Showed Preferential Recognition of Env-C V3 and Tat Epitopes

ADCC responses targeting specific epitopes may be beneficial in HIV control, similar to that observed with specific CD8+ CTL responses. To identify epitopes recognized by the ADCC antibodies from the Indian LTNPs, we used a matrix approach using subpools of peptides. The peptide pools in the matrix were designed in such a way that a single peptide was common in both the pools. ADCC epitopes identified in the matrix approach were then confirmed by NK cell activation assay using the single peptide present in both the pools to which the response was observed. We studied epitopes from Env-C and Tat antigens, as the responses to these antigens were associated with lower plasma viral load and also were higher in frequencies and magnitudes in the LTNP cohort. Due to the scarcity of the samples from the progressor group, we mapped the potential ADCC epitopes only in LTNPs and only the frequently recognized peptides (when recognized by 3 or more than 3 LTNPs) were tested in progressors.

The consensus Env-C overlapping peptides were 212 in number and the number of consensus Tat B peptides were 23. Out of 212 Env-C peptides, 153 peptides were recognized by ADCC antibodies from 10 LTNPs and 16 out of 23 Tat B peptides were recognized by 3 LTNPs (Table 2). One LTNP showed response to Env and Tat peptides. It was observed that the conserved C1, C2, C4, and C5 region and the variable V3, V4, and V5 and a few CD4 binding sites were recognized by the ADCC antibodies from Indian LTNPs (Table 2). In the gp41 region, both transmembrane and cytoplasmic tail regions were also identified. The Env-C V3 region (aa 288–330: LNESVEIVCTRPNNNTRKSIRIGPGQTGDIIGDIRQAHC) was preferentially identified by Indian LTNPs (5/10 Env-C responders) (Table 2). This region has also been previously identified by the monoclonal antibodies (694/98D, 4117C, 41148D, CH22, CH23) generated from the HIV-1 B-infected ADCC responders (19–22). None of the progressors identified this peptide (*p* = 0.0002 by Fisher’s exact test) (Table 3), indicating a potential role of ADCC antibodies recognizing this region in virus control. Additionally, Indian LTNPs also showed recognition of novel antigenic stretches from the signal peptide region (aa 1–15), CD4 binding sites (aa 113–117 and aa 361–366), C2 region (aa 250–288), aa C3 (aa 331–360), V4, C4, and V5 region (aa 381–470), and fusion peptide region of gp41 (aa 510–570) (Table 2). Three “Tat” regions (peptide no. 8, 12–13, 20–22) were recognized by three (8.82%) LTNPs. None of the progressors identified these regions (Fisher’s exact test: *p* < 0.05 for all) (Table 3).

**Table 2:** **Envelope (consensus HIV-1 C) peptides and tat (consensus HIV-1 B) regions recognized by Indian long-term non-progressors (LTNPs)**.

LTNP number	Env-C		Tat B	
	No of peptides recognized	Protein region	No of peptides recognized	Protein region
LTNP 8	0		4	Nuclear localization region
LTNP 10	30	C2–V3 region	–	
LTNP 14	4	V3, C3	–	
LTNP 23	17	C1, C3, V4, C5 cytoplasmic tail of gp41	–	
LTNP 30	1	V3	–	
LTNP 34	24	Signal peptide, C1 and C2	–	
LTNP 37	8	Fusion peptide region	–	
LTNP 50	0		11	Nuclear localization region
LTNP 45	1	V3	–	
LTNP 48	4	C1, V3	–	
LTNP 54	2	C1	–	
LTNP 58	66	C1, V3 C3 region with Cd4 binding site in C3, V4, C4, C5 fusion peptide region cytoplasmic tail and cytoplasmic tail in gp41 region	5	Nuclear
				Localization
				Region

**Table 3:** **Comparison of envelope C peptides and tat B regions recognized by Indian long-term non-progressors (LTNPs)**.

HIV-1 protein	Peptide no.*	Sequence	No. responder/no tested (% of response)		Fisher’s exact test
			LTNP	Progressor	
Envelop C (consensus HIV-1 C)	E-6	FWMLMICNVMGNLWV	1/34 (2.9%)	0/58	0.369 (NS)
	E12	KEAKTTLFCASDAKA	1/34 (2.9%)	0/58	0.369 (NS)
	E-73–80	LNESVEIVCTRPNNNTRKSIRIGPGQTGDIIGDIRQAHC	8/34 (23.5%)	0/58	0.0002
Tat (consensus HIV-1 B)	T-8	KCCFHCQVCFITKGL	3/34 (8.82%)	0/58	0.0477
	T-12, 13	ISYGRKKRRQRRRAPQDSQ			
	T-20, 21, 22	PRGDPTGPKESKKKVERETETDP			

## Discussion

Understanding the immune responses controlling HIV replication and disease progression will be critical in the development of improved preventive and curative strategies. LTNPs and elite controllers can be considered as a model for slow disease progression with better virus control. In addition to cellular immunity, there is emerging literature on the role of functional antibodies in virus control in these populations (23, 24). Ackerman and colleagues recently showed that subtype B-infected elite controllers with spontaneous virus control have a combination of various antibody effector mechanisms, including ADCC (25). Our study showed that the anti-Env ADCC responses were significantly higher in LTNPs with low plasma viral load (<2,000 copies/ml), suggesting a probable role for ADCC in controlling viral replication in subtype C HIV infections in India. We also found that ADCC antibodies from LTNPs were significantly more likely to identify more than one antigen simultaneously compared to progressors. Broad CTL responses have been shown to be associated with slow or no progression (26–28). It might be possible that broad ADCC responses limit opportunities for immune escape from ADCC reducing the disease progression (9). It will be interesting to estimate ADCC responses to various epitopes over time in this cohort to confirm this hypothesis.

Further characterization of anti-Env and anti-Tat ADCC responses in our Indian LTNP cohort found that they were associated with higher CD4 counts and lower plasma viral loads at the time of study visit and also with lower median plasma viral load calculated for the entire follow-up period. Similar findings have been reported by other groups studying subjects infected with HIV-1 subtypes B and A (5, 29–31), suggesting that the role of ADCC responses in controlling viral multiplication holds true across multiple clades.

The envelope protein is frequently targeted by ADCC antibodies from subtype B-infected individuals and non-human primates (22, 32–37). We have shown that ADCC responses in Indian LTNPs also preferentially target Env-C region, confirming a key role for Env-specific ADCC responses in virus control. The use of overlapping peptide pools allowed us to map Env-specific ADCC responses to individual linear peptide epitopes. We found ADCC antibodies from Indian LTNPs recognized both variable and conserved regions of Env-C antigens. Different ADCC monoclonal antibodies generated from HIV-1 subtype B infected population were shown to be specific to CD4-induced epitopes (19, 38). In our LTNP cohort, we found that the peptides spanning the V3 region of Env were commonly targeted (5/10 of Env-C responders), indicating that the antibodies recognizing HIV-1 Env region independent of CD4 binding region might also be important in viral control. Indeed, in our study, four of the five LTNPs who recognized this epitope have undetectable plasma viral load (Figure 3E). The main caveat of the anti-variable loop ADCC responses is the variability in this region across viruses. However, detection of these responses in HIV-1 LTNPs emphasizes a potential role of anti-V3 ADCC responses in viral control in HIV-1 C-infected patients. Effective V3-specific ADCC antibodies could also have neutralizing capacity, providing an additional level of immune control (39). Additional research is required to know whether these antibodies also have a role in clearing of latent reservoirs where the virus can downregulate CD4, making it inaccessible for CD4-dependent ADCC antibodies. It was also observed that a few previously identified regions such as V2 region of gp120 and immunodominant region of gp41 were not recognized by Indian LTNPs (22). This differential recognition of ADCC epitopes by individuals infected with different subtypes has implications for vaccine designs and immunotherapeutic strategies. The importance of recognition of these epitopes could be assessed in longitudinal samples to determine whether the sustained recognition of these regions influences disease progression.

Our finding that Indian LTNPs develop Tat-specific ADCC responses was novel and unexpected although only 4 of 34 LTNPs showed Tat-specific response. A number of researchers have reported ADCC responses against Nef, Vpu, and Pol (7, 40, 41), but in our cohort, we found significantly higher and more common recognition of Tat peptides in LTNPs. Tat is one of the earliest expressed proteins in HIV-1 infection and is responsible for efficient transcription (42–45). It has been shown that the presence of Tat-specific CTLs and/or anti-Tat antibody is associated with control of viral replication and slow progression to AIDS (46–48). In non-human primate models, greater protection was correlated with Tat- and Env-binding antibodies along with higher ADCC and ADCVI activities (49). The ADCC antibodies generated against the antigen expressed early in the life cycle might be able to control viral replication early. It will be interesting to study Tat-specific monoclonal and polyclonal antibodies to determine whether antibodies generated against individual Tat epitopes can lyse HIV-infected cells and control HIV infection *in vitro*. We have used subtype B Tat peptides in the study as subtype C Tat peptides were not available. This is the limitation of the study. However, we carried out a blast analysis of Tat sequences of consensus subtype C and consensus subtype B, which showed that the three Tat epitope regions identified by Indian LTNPs had 69, 93, and 82% similarity (Figure S2 in Supplementary Material). Given our findings, future studies using consensus subtype C Tat peptides or autologous peptide sets based on individual viruses are now warranted.

Since the ADCC activity observed in the NK cell activation assays significantly correlate with a functional killing assay (11), we can speculate that NK cell activating antibodies from Indian LTNPs would have an ability to kill HIV-infected cells *in vivo*; future studies are needed to study the efficiency of target cell killing by these antibodies.

In conclusion, ADCC responses have been characterized in Indian LTNPs for the first time. The findings added to the global data and confirm the association of broad ADCC responses in viral containment across different subtypes. The frequently recognized peptides from the V3 loop of Env-C and Tat antigen by LTNPs with controlled viral replication warrants further studies to understand their probable role in clearing the latent infection and also in HIV protection.

## Ethics Statement

The study design was approved by an institutional ethics review board of National AIDS Research Institute. All study participants provided informed consent before the collection of blood samples. The study did not involve any vulnerable population.

## Author Contributions

AK performed the research, data analyses, and wrote the manuscript. MT and SJK designed the study and commented on manuscript. RP commented on manuscript. AS, SK, and VM performed parts of the research and helped in manuscript writing. MG and SG contributed clinical information and samples for the study.

## Conflict of Interest Statement

The authors declare that the research was conducted in the absence of any commercial or financial relationships that could be construed as a potential conflict of interest.

## References

[1] Haynes BF Gilbert PB McElrath MJ Zolla-Pazner S Tomaras GD Alam SM Immune-correlates analysis of an HIV-1 vaccine efficacy trial. N Engl J Med (2012) 366(14):1275–86. 10.1056/NEJMoa1113425 22475592 PMC3371689

[2] Ljunggren K Moschese V Broliden PA Giaquinto C Quinti I Fenyo EM Antibodies mediating cellular cytotoxicity and neutralization correlate with a better clinical stage in children born to human immunodeficiency virus-infected mothers. J Infect Dis (1990) 161(2):198–202. 10.1093/infdis/161.2.198 2299204

[3] Mandalia S Westrop SJ Beck EJ Nelson M Gazzard BG Imami N. Are long-term non-progressors very slow progressors? Insights from the Chelsea and Westminster HIV cohort, 1988-2010. PLoS One (2012) 7(2):e29844. 10.1371/journal.pone.0029844 22363409 PMC3282685

[4] Scheid JF Mouquet H Feldhahn N Seaman MS Velinzon K Pietzsch J Broad diversity of neutralizing antibodies isolated from memory B cells in HIV-infected individuals. Nature (2009) 458(7238):636–40. 10.1038/nature07930 19287373

[5] Lambotte O Ferrari G Moog C Yates NL Liao HX Parks RJ Heterogeneous neutralizing antibody and antibody-dependent cell cytotoxicity responses in HIV-1 elite controllers. AIDS (2009) 23(8):897–906. 10.1097/QAD.0b013e328329f97d 19414990 PMC3652655

[6] Blankson JN. Effector mechanisms in HIV-1 infected elite controllers: highly active immune responses? Antiviral Res (2010) 85(1):295–302. 10.1016/j.antiviral.2009.08.007 19733595 PMC2814919

[7] Wren LH Chung AW Isitman G Kelleher AD Parsons MS Amin J Specific antibody-dependent cellular cytotoxicity responses associated with slow progression of HIV infection. Immunology (2013) 138(2):116–23. 10.1111/imm.12016 23173935 PMC3575764

[8] Kulkarni AG Paranjape RS Thakar MR. Higher expression of activating receptors on cytotoxic NK cells is associated with early control on HIV-1C multiplication. Front Immunol (2014) 5:222. 10.3389/fimmu.2014.00222 24904577 PMC4032894

[9] Chung AW Isitman G Navis M Kramski M Center RJ Kent SJ Immune escape from HIV-specific antibody-dependent cellular cytotoxicity (ADCC) pressure. Proc Natl Acad Sci U S A (2011) 108(18):7505–10. 10.1073/pnas.1016048108 21502492 PMC3088575

[10] Stratov I Chung A Kent SJ. Robust NK cell-mediated human immunodeficiency virus (HIV)-specific antibody-dependent responses in HIV-infected subjects. J Virol (2008) 82(11):5450–9. 10.1128/JVI.01952-07 18353957 PMC2395196

[11] Chung AW Rollman E Center RJ Kent SJ Stratov I. Rapid degranulation of NK cells following activation by HIV-specific antibodies. J Immunol (2009) 182(2):1202–10. 10.4049/jimmunol.182.2.1202 19124764

[12] Fischer L Penack O Gentilini C Nogai A Muessig A Thiel E The anti-lymphoma effect of antibody-mediated immunotherapy is based on an increased degranulation of peripheral blood natural killer (NK) cells. Exp Hematol (2006) 34(6):753–9. 10.1016/j.exphem.2006.02.015 16728280

[13] Bruckheimer EM Fazenbaker CA Gallagher S Mulgrew K Fuhrmann S Coffman KT Antibody-dependent cell-mediated cytotoxicity effector-enhanced EphA2 agonist monoclonal antibody demonstrates potent activity against human tumors. Neoplasia (2009) 11(6):509–17, 2 following 17. 10.1593/neo.81578 19484140 PMC2685440

[14] Bologna L Gotti E Manganini M Rambaldi A Intermesoli T Introna M Mechanism of action of type II, glycoengineered, anti-CD20 monoclonal antibody GA101 in B-chronic lymphocytic leukemia whole blood assays in comparison with rituximab and alemtuzumab. J Immunol (2015) 186(6):3762–9. 10.4049/jimmunol.1000303 21296976

[15] Lichtfuss GF Meehan AC Cheng WJ Cameron PU Lewin SR Crowe SM HIV inhibits early signal transduction events triggered by CD16 cross-linking on NK cells, which are important for antibody-dependent cellular cytotoxicity. J Leukoc Biol (2011) 89(1):149–58. 10.1189/jlb.0610371 20884651

[16] Sun Y Asmal M Lane S Permar SR Schmidt SD Mascola JR Antibody-dependent cell-mediated cytotoxicity in simian immunodeficiency virus-infected rhesus monkeys. J Virol (2011) 85(14):6906–12. 10.1128/JVI.00326-11 21593181 PMC3126600

[17] Alter G Malenfant JM Altfeld M. CD107a as a functional marker for the identification of natural killer cell activity. J Immunol Methods (2004) 294(1–2):15–22. 10.1016/j.jim.2004.08.008 15604012

[18] Thakar MR Bhonge LS Lakhashe SK Shankarkumar U Sane SS Kulkarni SS Cytolytic T lymphocytes (CTLs) from HIV-1 subtype C-infected Indian patients recognize CTL epitopes from a conserved immunodominant region of HIV-1 Gag and Nef. J Infect Dis (2005) 192(5):749–59. 10.1086/432547 16088824

[19] Williams KL Cortez V Dingens AS Gach JS Rainwater S Weis JF HIV-specific CD4-induced antibodies mediate broad and potent antibody-dependent cellular cytotoxicity activity and are commonly detected in plasma from HIV-infected humans. EBioMedicine (2015) 2(10):1464–77. 10.1016/j.ebiom.2015.09.001 26629541 PMC4634620

[20] Alsmadi O Tilley SA. Antibody-dependent cellular cytotoxicity directed against cells expressing human immunodeficiency virus type 1 envelope of primary or laboratory-adapted strains by human and chimpanzee monoclonal antibodies of different epitope specificities. J Virol (1998) 72(1):286–93. 9420226 10.1128/jvi.72.1.286-293.1998 PMC109375

[21] Bonsignori M Pollara J Moody MA Alpert MD Chen X Hwang KK Antibody-dependent cellular cytotoxicity-mediating antibodies from an HIV-1 vaccine efficacy trial target multiple epitopes and preferentially use the VH1 gene family. J Virol (2012) 86(21):11521–32. 10.1128/JVI.01023-12 22896626 PMC3486290

[22] Pollara J Bonsignori M Moody MA Pazgier M Haynes BF Ferrari G. Epitope specificity of human immunodeficiency virus-1 antibody dependent cellular cytotoxicity [ADCC] responses. Curr HIV Res (2013) 11(5):378–87. 10.2174/1570162X113116660059 24191939 PMC3878369

[23] Chung AW Navis M Isitman G Wren L Silvers J Amin J Activation of NK cells by ADCC antibodies and HIV disease progression. J Acquir Immune Defic Syndr (2011) 58(2):127–31. 10.1097/QAI.0b013e31822c62b9 21792067 PMC3175260

[24] Zhou T Georgiev I Wu X Yang ZY Dai K Finzi A Structural basis for broad and potent neutralization of HIV-1 by antibody VRC01. Science (2010) 329(5993):811–7. 10.1126/science.1192819 20616231 PMC2981354

[25] Ackerman ME Mikhailova A Brown EP Dowell KG Walker BD Bailey-Kellogg C Polyfunctional HIV-specific antibody responses are associated with spontaneous HIV control. PLoS Pathog (2016) 12(1):e1005315. 10.1371/journal.ppat.1005315 26745376 PMC4706315

[26] Borrow P Lewicki H Hahn BH Shaw GM Oldstone MB. Virus-specific CD8+ cytotoxic T-lymphocyte activity associated with control of viremia in primary human immunodeficiency virus type 1 infection. J Virol (1994) 68(9):6103–10. 8057491 10.1128/jvi.68.9.6103-6110.1994 PMC237022

[27] Betts MR Krowka JF Kepler TB Davidian M Christopherson C Kwok S Human immunodeficiency virus type 1-specific cytotoxic T lymphocyte activity is inversely correlated with HIV type 1 viral load in HIV type 1-infected long-term survivors. AIDS Res Hum Retroviruses (1999) 15(13):1219–28. 10.1089/088922299310313 10480635

[28] Koup RA Safrit JT Cao Y Andrews CA McLeod G Borkowsky W Temporal association of cellular immune responses with the initial control of viremia in primary human immunodeficiency virus type 1 syndrome. J Virol (1994) 68(7):4650–5. 8207839 10.1128/jvi.68.7.4650-4655.1994 PMC236393

[29] Johansson SE Rollman E Chung AW Center RJ Hejdeman B Stratov I NK cell function and antibodies mediating ADCC in HIV-1-infected viremic and controller patients. Viral Immunol (2011) 24(5):359–68. 10.1089/vim.2011.0025 21958370

[30] Baum LL Cassutt KJ Knigge K Khattri R Margolick J Rinaldo C HIV-1 gp120-specific antibody-dependent cell-mediated cytotoxicity correlates with rate of disease progression. J Immunol (1996) 157(5):2168–73. 8757343

[31] Ahmad R Sindhu ST Toma E Morisset R Vincelette J Menezes J Evidence for a correlation between antibody-dependent cellular cytotoxicity-mediating anti-HIV-1 antibodies and prognostic predictors of HIV infection. J Clin Immunol (2001) 21(3):227–33. 10.1023/A:1011087132180 11403230

[32] Lyerly HK Reed DL Matthews TJ Langlois AJ Ahearne PA Petteway SR Jr Anti-GP 120 antibodies from HIV seropositive individuals mediate broadly reactive anti-HIV ADCC. AIDS Res Hum Retroviruses (1987) 3(4):409–22. 10.1089/aid.1987.3.409 2833917

[33] Tyler DS Stanley SD Nastala CA Austin AA Bartlett JA Stine KC Alterations in antibody-dependent cellular cytotoxicity during the course of HIV-1 infection. Humoral and cellular defects. J Immunol (1990) 144(9):3375–84. 2329275

[34] Koup RA Robinson JE Nguyen QV Pikora CA Blais B Roskey A Antibody-dependent cell-mediated cytotoxicity directed by a human monoclonal antibody reactive with gp120 of HIV-1. AIDS (1991) 5(11):1309–14. 10.1097/00002030-199111000-00004 1722676

[35] Pegu P Vaccari M Gordon S Keele BF Doster M Guan Y Antibodies with high avidity to the gp120 envelope protein in protection from simian immunodeficiency virus SIV(mac251) acquisition in an immunization regimen that mimics the RV-144 Thai trial. J Virol (2013) 87(3):1708–19. 10.1128/JVI.02544-12 23175374 PMC3554145

[36] Schifanella LGS Vaccari M Binello N Caccuri F Blackburn M Fenizia C, editors. MF59 and ALUM, in combination with an ALVAC-SIV/gp120 vaccine, induce plasmablasts that differ in the expression of homing markers. AIDS Vaccine. Barcelona, Spain: AIDS Research and Human retroviruses (2013) p. A13.

[37] Gomez-Roman VR Patterson LJ Venzon D Liewehr D Aldrich K Florese R Vaccine-elicited antibodies mediate antibody-dependent cellular cytotoxicity correlated with significantly reduced acute viremia in rhesus macaques challenged with SIVmac251. J Immunol (2005) 174(4):2185–9. 10.4049/jimmunol.174.4.2185 15699150

[38] Veillette M Desormeaux A Medjahed H Gharsallah NE Coutu M Baalwa J Interaction with cellular CD4 exposes HIV-1 envelope epitopes targeted by antibody-dependent cell-mediated cytotoxicity. J Virol (2014) 88(5):2633–44. 10.1128/JVI.03230-13 24352444 PMC3958102

[39] Bruel T Guivel-Benhassine F Amraoui S Malbec M Richard L Bourdic K Elimination of HIV-1-infected cells by broadly neutralizing antibodies. Nat Commun (2016) 7:10844 10.1038/ncomms10844 26936020 PMC4782064

[40] Yamada T Watanabe N Nakamura T Iwamoto A. Antibody-dependent cellular cytotoxicity via humoral immune epitope of Nef protein expressed on cell surface. J Immunol (2004) 172(4):2401–6. 10.4049/jimmunol.172.4.2401 14764710

[41] Isitman G Stratov I Kent SJ. Antibody-dependent cellular cytotoxicity and NK cell-driven immune escape in HIV infection: implications for HIV vaccine development. Adv Virol (2012) 2012:637208. 10.1155/2012/637208 22611395 PMC3350948

[42] Arya SK Guo C Josephs SF Wong-Staal F. Trans-activator gene of human T-lymphotropic virus type III (HTLV-III). Science (1985) 229(4708):69–73. 10.1126/science.2990040 2990040

[43] Fisher AG Feinberg MB Josephs SF Harper ME Marselle LM Reyes G The trans-activator gene of HTLV-III is essential for virus replication. Nature (1986) 320(6060):367–71. 10.1038/320367a0 3007995

[44] Ensoli B Cafaro A HIV-1 Tat vaccines. Virus Res (2002) 82(1–2):91–101. 10.1016/S0168-1702(01)00393-8 11885958

[45] Goldstein G HIV-1 Tat protein as a potential AIDS vaccine. Nat Med (1996) 2(9):960–4. 10.1038/nm0996-960 8782444

[46] Re MC Furlini G Vignoli M Ramazzotti E Roderigo G De Rosa V Effect of antibody to HIV-1 Tat protein on viral replication in vitro and progression of HIV-1 disease in vivo. J Acquir Immune Defic Syndr Hum Retrovirol (1995) 10(4):408–16. 10.1097/00042560-199512000-00003 7583436

[47] Addo MM Altfeld M Rosenberg ES Eldridge RL Philips MN Habeeb K The HIV-1 regulatory proteins Tat and Rev are frequently targeted by cytotoxic T lymphocytes derived from HIV-1-infected individuals. Proc Natl Acad Sci U S A (2001) 98(4):1781–6. 10.1073/pnas.98.4.1781 11172028 PMC29334

[48] Zagury D Lachgar A Chams V Fall LS Bernard J Zagury JF Interferon alpha and Tat involvement in the immunosuppression of uninfected T cells and C-C chemokine decline in AIDS. Proc Natl Acad Sci U S A (1998) 95(7):3851–6. 10.1073/pnas.95.7.3851 9520456 PMC19926

[49] Florese RH Demberg T Xiao P Kuller L Larsen K Summers LE Contribution of nonneutralizing vaccine-elicited antibody activities to improved protective efficacy in rhesus macaques immunized with Tat/Env compared with multigenic vaccines. J Immunol (2009) 182(6):3718–27. 10.4049/jimmunol.0803115 19265150 PMC2744397

