# Supplementary material for: Indian Long-term Non-Progressors Show Broad ADCC Responses with Preferential Recognition of V3 Region of Envelope and a Region from Tat Protein
Source: Front Immunol. 2017 Jan 19;8:5. doi: 10.3389/fimmu.2017.00005 (PMC5243827; doi:10.3389/fimmu.2017.00005)
Supplement: Supplementary file 1 [file Data_Sheet_1.DOCX]

Supplementary Material

**Indian Long Term Non Progressors show broad ADCC responses with preferential recognition of V3 region of Envelop and a region from Tat protein**

Archana Kulkarni^1^, Swarali Kurle^1^, Ashwini Shete^1^, Manisha Ghate^2^, Sheela Godbole^3^, Vijaya Madhavi^4^, Stephen J Kent^4^, Ramesh Paranjape^1^ and Madhuri Thakar^1^*

*** Correspondence: Dr. Madhuri Thakar-** [mthakar@nariindia.org](mailto:mthakar@nariindia.org),

| Sr No | Name | Cat No | No of peptides |
| --- | --- | --- | --- |
| 1 | HIV-1 Consensus C Env Peptide Set (15 mer overlapping peptides) | 9499 | 212 |
| 2 | HIV-1 Consensus B Env Peptide Set (15 mer overlapping peptides) | 9480 | 211 |
| 3 | HIV-1 Consensus B Pol Peptide Set (15 mer overlapping peptides) | 6208 | 249 |
| 4 | HIV-1 Consensus B Tat Peptide Set (15 mer overlapping peptides) | 5138 | 23 |
| 5 | HIV-1 Consensus B Rev Peptide Set (15 mer overlapping peptides) | 6445 | 27 |
| 6 | HIV-1 Consensus B Nef Peptide Set (15 mer overlapping peptides) | 5189 | 49 |
| 7 | HIV-1 Consensus B Vpu Peptide Set (15 mer overlapping peptides) | 6444 | 19 |
| 8 | HIV-1 Subtype C (96ZM651.8) Gag Peptide Set(20 mer overlapping peptides) | 3993 | 49 |

**Supplementary Table-1: Details of HIV-1 antigen peptide sets used in NK cell activation assay.**

**Preparation of peptide pools**-Fifteen amino acid long peptides for HIV-1 antigens [Env (subtypes B &C), ,Pol ,Tat, Rev, Nef & Vpu(subtype B)] overlapping by 11 amino acids and 20 mer overlapping peptides in case of HIV-1 Gag C were provided by the National Institutes of Health AIDS reagent repository. For each HIV-1 antigenic peptide pools, complete sets of peptides were solubilized in DMSO (at 20μg/μl) and pooled to a single pool ( stock concentration of 80μg/ml)for evaluation of ADCC responses at afinal concentration of 1μg/ml.To identify and map ADCC activity across Env C and Tat B, we prepared30 matrix pools for env C and 10 matrix pools for HIV-1 Tat B. (as shown in figure-1 A and figure -1B in such a way that each peptide is repeated in two pools. (stock concentration of 80μg/ml ) The final working concentration of each peptide in the assay was kept constant i.e. 1μg/ml.

**Supplementary Figure-1A &B** Matrix peptide pools for Consensus envelope C(A) and tat B(B)( These pools were prepared in such a way that each peptide is repeated in two pools. (ref- Thakar MR, Bhonge LS, Lakhashe SK, Shankarkumar U, Sane SS, Kulkarni SS, et al. Cytolytic T lymphocytes (CTLs) from HIV-1 subtype C-infected Indian patients recognize CTL epitopes from a conserved immunodominant region of HIV-1 Gag and Nef. *J Infect Dis* (2005) 192(5):749-59. PubMed PMID: 16088824)

**[A] HIV-1 C Envelope**

|  | **Pool# 1** | **Pool# 2** | **Pool# 3** | **Pool# 4** | **Pool# 5** | **Pool# 6** | **Pool# 7** | **Pool# 8** | **Pool# 9** | **Pool# 10** | **Pool# 11** | **Pool# 12** | **Pool# 13** | **Pool# 14** |
| --- | --- | --- | --- | --- | --- | --- | --- | --- | --- | --- | --- | --- | --- | --- |
| **Pool#15** | E-1 | E-2 | E-3 | E-4 | E-5 | E-6 | E-7 | E-8 | E-9 | E-10 | E-11 | E-12 | E-13 | E-14 |
| **Pool#16** | E-15 | E-16 | E-17 | E-18 | E-19 | E-20 | E-21 | E-22 | E-23 | E-24 | E-25 | E-26 | E-27 | E-28 |
| **Pool#17** | E-29 | E-30 | E-31 | E-32 | E-33 | E-34 | E-35 | E-36 | E-37 | E-38 | E-39 | E-40 | E-41 | E-42 |
| **Pool#18** | E-43 | E-44 | E-45 | E-46 | E-47 | E-48 | E-49 | E-50 | E-51 | E-52 | E-53 | E-54 | E-55 | E-56 |
| **Pool#19** | E-57 | E-58 | E-59 | E-60  E-77 is present in both pools i.e. pool number-7 and pool number 20 (suggesting that if ADCC response is seen to both pools then E-77 peptide is responsible for the response | E-61 | E-62 | E-63 | E-64 | E-65 | E-66 | E-67 | E-68 | E-69 | E-70 |
| **Pool#20** | E-71 | E-72 | E-73 | E-74 | E-75 | E-76 | E-77 | E-78 | E-79 | E-80 | E-81 | E-82 | E-83 | E-84 |
| **Pool#21** | E-85 | E-86 | E-87 | E-88 | E-89 | E-90 | E-91 | E-92 | E-93 | E-94 | E-95 | E-96 | E-97 | E-98 |
| **Pool#22** | E-99 | E-100 | E-101 | E-102 | E-103 | E-104 | E-105 | E-106 | E-107 | E-108 | E-109 | E-110 | E-111 | E-112 |
| **Pool#23** | E-113 | E-114 | E-115 | E-116 | E-117 | E-118 | E-119 | E-120 | E-121 | E-122 | E-123 | E-124 | E-125 | E-126 |
| **Pool#24** | E-127 | E-128 | E-129 | E-130 | E-131 | E-132 | E-133 | E-134 | E-135 | E-136 | E-137 | E-138 | E-139 | E-140 |
| **Pool#25** | E-141 | E-142 | E-143 | E-144 | E-145 | E-146 | E-147 | E-148 | E-149 | E-150 | E-151 | E-152 | E-153 | E-154 |
| **Pool#26** | E-155 | E-156 | E-157 | E-158 | E-159 | E-160 | E-161 | E-162 | E-163 | E-164 | E-165 | E-166 | E-167 | E-168 |
| **Pool#27** | E-169 | E-170 | E-171 | E-172 | E-173 | E-174 | E-175 | E-176 | E-177 | E-178 | E-179 | E-180 | E-181 | E-182 |
| **Pool#28** | E-183 | E-184 | E-185 | E-186 | E-187 | E-188 | E-189 | E-190 | E-191 | E-192 | E-193 | E-194 | E-195 | E-196 |
| **Pool#29** | E-197 | E-198 | E-199 | E-200 | E-201 | E-202 | E-203 | E-204 | E-205 | E-206 | E-207 | E-208 | E-209 | E-210 |
| **Pool#30** | E-211 | E-212 |  |  |  |  |  |  |  |  |  |  |  |  |

**[B]HIV-1 Tat B**

|  | Pool# 1 | Pool# 2 | Pool# 3 | Pool# 4 | Pool# 5 |
| --- | --- | --- | --- | --- | --- |
| Pool#6  T-20 is present in both pools i.e. pool number-5 and pool number 9 (suggesting that if ADCC response is seen to both pools then T-20 peptide is responsible for the response | T-1 | T-2 | T-3 | T-4 | T-5 |
| Pool#7 | T-6 | T-7 | T-8 | T-9 | T-10 |
| Pool#8 | T-11 | T-12 | T-13 | T-14 | T-15 |
| Pool#9 | T-16 | T-17 | T-18 | T-19 | T-20 |
| Pool#10 | T-21 | T-22 | T-23 |  |  |

**Table 2: Distribution of CD107a and IFN-γ expression by ADCC mediated activated NK cells in responders from both groups against various HIV-1 antigens**

| **Structural proteins** |  | **Env B** | | | | **Env C** | | | **Gag C** | | | **Pol B** | | |
| --- | --- | --- | --- | --- | --- | --- | --- | --- | --- | --- | --- | --- | --- | --- |
| Study groups |  | **Only CD107a** | **Both (CD107a & IFN-γ)** | **Only IFN-γ** | | **Only CD107a** | **Both (CD107a & IFN-γ)** | **Only IFN-γ** | **Only CD107a** | **Both (CD107a & IFN-γ)** | **Only IFN-γ** | **Only CD107a** | **Both (CD107a & IFN-γ)** | **Only IFN-γ** |
| LTNPs | Mean | 2.27 | 1.66 | 5.43 | | 4.56 | 4 | 8.10 | 1.40 | 1.27 | 5.09 | No responder | | |
|  | Range- | 0-8.64 | 0-4.91 | 1.75-9.15 | | 0-11.51 | 0-8.34 | 2.02-17.44 | 0-3.00 | 0.18-2.81 | 0.36-9.63 |  |  |  |
| Progressors | Mean | 3.19 | 2.47 | 4.40 | | 4.49 | 0.59 | 1.25 | No responder | | | 8.2 | 6.38 | 8.39 |
|  | Range- | 0.23-10.83 | 0-8.01 | 0-17.11 | | 0.53-9.54 | 0.52-7.06 | 2.64-8.29 |  |  |  | Only one responder | | |
| **Accessory proteins** |  | **Tat B** | | | | **Rev B** | | | **Nef B** | | | **Vpu B** | | |
| Study groups |  | **Only CD107a** | **Both (CD107a & IFN-γ)** | | **Only IFN-γ** | **Only CD107a** | **Both (CD107a & IFN-γ)** | **Only IFN-γ** | **Only CD107a** | **Both (CD107a & IFN-γ)** | **Only IFN-γ** | **Only CD107a** | **Both (CD107a & IFN-γ)** | **Only IFN-γ** |
| LTNPs | Mean | 1.46 | 0.72 | | 7.79 | 0.49 | 0.30 | 5.82 | 1.08 | 1.10 | 5.20 | 0.49 | 0.045 | 4.55 |
|  | Range- | 0-5.53 | 0-2.52 | | 0.052-14.13 | 0.49-1.71 | 0.30-1.2 | 1.62-8.44 | 0-2.99 | 0.32-3.25 | 2.60-9.62 | Only one responder | | |
| Progressors | Mean | 1.58 | 0.712 | | 1.99 | 2.38 | 1.41 | 4.62 | 1.84 | 0.84 | 2.12 | 3.96 | 3.40 | 5.04 |
|  | Range- | Only one responder | | | | 1.55-2.97 | 1-2.05 | 0.71-11.96 | 0.034-4.31 | 0-2.4 | 0-4.53 | 0-7.25 | 0.625-6.255 | 2.98-7.75 |

**
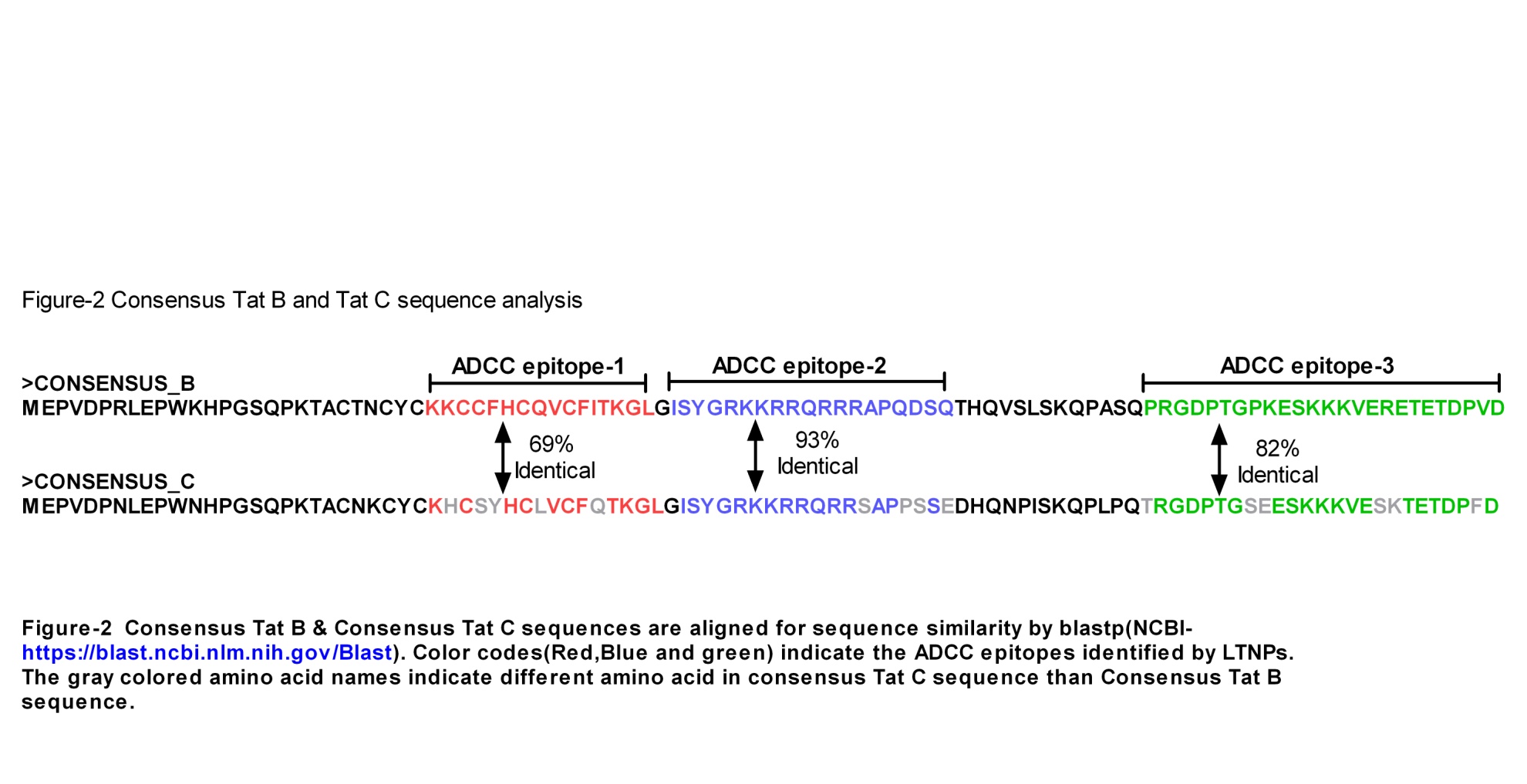
**
